# Synthesis and Biological Activities of Pyrazino[1,2-*a*]indole and Pyrazino[1,2-*a*]indol-1-one Derivatives

**DOI:** 10.3390/ph14080779

**Published:** 2021-08-08

**Authors:** Kena Zhang, Christine Tran, Mouad Alami, Abdallah Hamze, Olivier Provot

**Affiliations:** BioCIS, CNRS-Université Paris-Saclay, 92290 Châtenay-Malabry, France; kena.zhang@universite-paris-saclay.fr (K.Z.); christine.tran@universite-paris-saclay.fr (C.T.); mouad.alami@universite-paris-saclay.fr (M.A.); abdallah.hamze@universite-paris-saclay.fr (A.H.)

**Keywords:** pyrazinoindole, pyrazinoindolone, cyclization, catalysis, biological activity

## Abstract

This review concerns the synthesis and biological activities of pyrazino[1,2-*a*]indoles and pyrazino[1,2-*a*]indol-1-ones reported since 1997 and the discovery of biological activity of pyrazinoindole derivatives. In the first part, we first presented the synthetic routes that have been reported from a methodological point of view to access the pyrazinoindole unit according to cyclization reactions using or not using metal catalysts. Then, syntheses and neuropsychiatric, auto-immune, anti-infectious and anti-cancer properties of pyrazinoindoles were detailed. In the second part, we first reported the main accesses to pyrazinoindol-1-one substrates according to Michael reactions, metal-catalyzed and metal-free cyclization reactions. The syntheses and anti-cancer, anti-infectious, anti-allergenic and neuropsychiatric properties of pyrazinoindolones were next described and discussed.

## 1. Introduction

**Figure 1:**
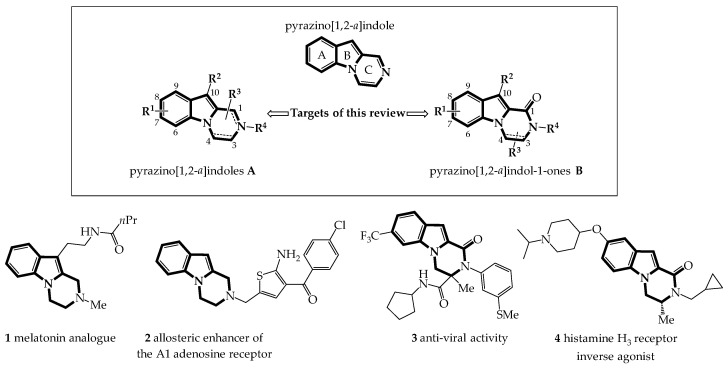
Targets of this review and selection of biologically active tetrahydro-pyrazino[1,2-*a*]indoles **1a**, **2a** and dihydro-pyrazino[1,2-*a*]indol-1-ones **3a**, **4a**.

The pyrazino[1,2-*a*]indole unit is a tricyclic aromatic nucleus combining an indole and a pyrazine linked by the N5 and C9a atoms (Figure 1).

The access to this substituted aromatic nucleus has been well studied since 1997 by the chemist community from a synthetic point of view and for its potential in medicinal chemistry [1,2]. In parallel, structural modifications of the pyrazino[1,2-*a*]indole nucleus showed that (3,4-dihydro)pyrazino[1,2-*a*]indoles (type A) and (3,4-dihydro)-pyrazino[1,2-*a*]indol-1-ones (type B) were efficient pharmacophores used in a variety of diseases. To illustrate, 3,4-dihydropyrazinoindoles **1** [3] and **2** [4] (type A) have been showed to be effective at melatonin and adenosine receptors, while 3,4-dihydropyrazinoindol-1-ones **3** [5] and **4** [6] (type B) have been studied for their anti-viral and anti-allergenic activities, respectively. This review, which follows recent papers from our group dealing with the synthesis of indole-fused heterocycles such as pyrido[1,2-*a*]indoles [7] and oxazino[4,3-*a*]indoles [8] is divided into two parts. First, we compiled the recent syntheses and biological activities of pyrazinoindoles A and then discussed on the biological properties of pyrazinoindol-1-ones B and on their preparation. It should be noted that patents dealing with this subject have not been mentioned in this review. Each part is preceded by a short introduction dealing with recent synthetic accesses to these types of heterocycles that have not been studied biologically.

## 2. Pyrazinoindoles A: Synthesis and Biological Properties

### 2.1. Recent Synthetic Approaches to Variously Substituted Pyrazinoindoles and 3,4-Dihydropyrazinoindoles

**Scheme 1:**
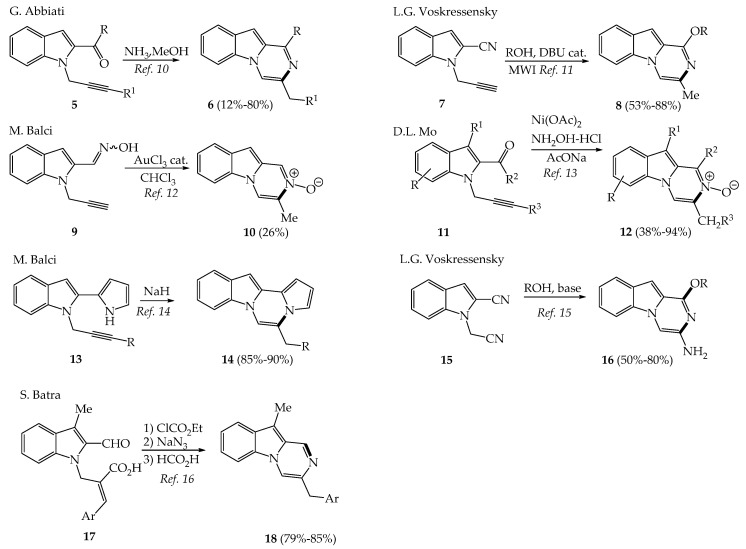
Synthesis of pyrazinoindoles.

The creation of the pyrazino[1,2-*a*]indole nucleus was mainly achieved by cyclizing indole having various groups (CHO, ketone, imine, nitrile, etc.) on C2 with a nucleophile linked to the indole nitrogen atom, thus creating the pyrazino C-ring (Scheme 1). For example, 2-substituted-1-(prop-2-yn-1-yl)-1*H*-indoles **5**, **7**, **9**, **11**, **13** transformed into pyrazinoindoles **6**, **8**, **10**, **12** and **14** respectively by intramolecular cyclization using NH_3_ in MeOH, [9,10] DBU under microwave irradiation, [11] AuCl_3_ [12] as triple bond activator, Ni(OAc)_2_ in the presence of hydroxylamine [13] or NaH in DMF [14].

The C-ring of the pyrazino[1,2-*a*]indole system has been also built by alcoholate promoted cyclization of indolodinitrile compound **15** [15] and by Curtius reaction using Morita–Baylis–Hillman derivatives **17** [16] with good to excellent yields.

**Scheme 2:**
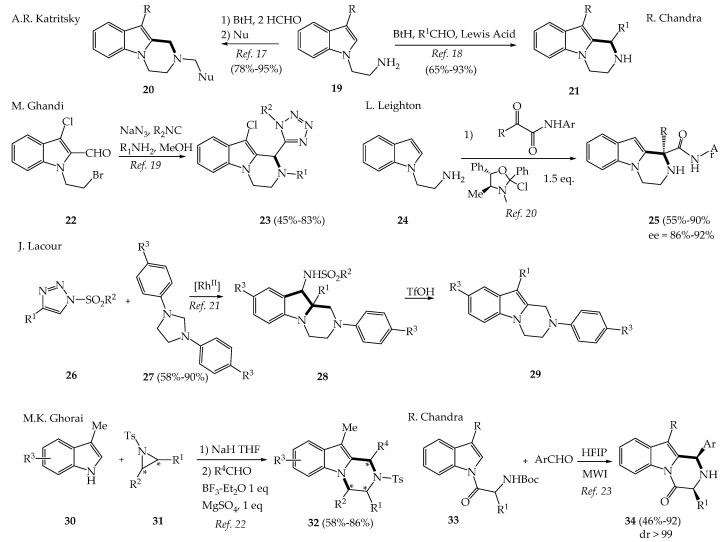
Synthesis of 1,2,3,4-tetrahydro-pyrazinoindoles.

The synthesis of variously substituted pyrazinoindoles having a saturated C-ring has been more studied than that of their aromatic counterparts, probably because these compounds offer more functional diversity such as diastereoselective accesses, but mainly because they have shown superior efficacy in medicinal chemistry. Among the simplest reactions described to prepare these compounds was the one proposed by Katritzky, who used a cycloaddition reaction between a *N*-ethylamine-indole **19** and formaldehyde in the presence of benzotriazole (Bt) (Scheme 2). A subsequent nucleophilic substitution reaction of the benzotriazole gives rise to various *N*-substituted pyrazinoindoles **20** [17]. *N*-ethylamine-indoles **19** also reacted, in a complementary approach, with aldehydes and Bt in the presence of Lewis acids to give C1-substituted pyrazinoindoles **21** [18].

A Ugi-azide four component approach was recently published to prepare a series of *N*-substituted pyrazinoindoles **23** having on C1 a substituted tetrazole ring [19]. Leighton et al. proposed highly enantioselective iso-Pictet–Spengler reactions using the condensation of 2-(1*H*-indol-1-yl)ethanamine **24** with a variety of α-ketoamides, followed by the addition of a commercially available chiral silicon Lewis acid (L*) to give 1,1-disubstituted-tetrahydropyrazino[1,2-*a*]indoles **25** with good yields (55–90%) and high enantioselectivity (ee = 86–96%) [20]. Guinchard et al. also reported an Au(I)-catalyzed Pictet–Spengler reaction to prepare a variety of complex heterocyclic compounds including tetrahydro-pyrazinoindoles with good yields ranging from 43 to 93% [21]. In 2021, Lacour et al. reported that *N*-sulfonyltriazoles **26** and imidazolines **27** reacted under rhodium catalysis to give a variety of hexahydro-pyrazinoindoles **28** with excellent yields easily transformed in tetrahydropyrazinoindoles **29** after a welcome rearrangement in triflic acid TfOH [22]. Ghorai et al. reported in 2018 of an elegant synthesis of 1,3-disubstituted 1,2,3,4-tetrahydropyrazino[1,2-*a*]indoles **32** with excellent stereoselectivity (de, ee >99%) via base-mediated ring opening of chiral aziridines **31** with skatoles **30** followed by BF_3_-OEt_2_ catalyzed Pictet–Spengler reaction [23]. Chandra group reported synthesis of di-substituted pyrazinoindol-4-ones **34** with an excellent diastereoselectivity (>99%) via a Pictet–Spengler reaction by mixing 3-substituted-*N*-acylindoles **33** and aromatic aldehydes in the presence of hexafluoroisopropanol (HFIP) under microwave irradiation [24].

After this overview of recent methodologies giving access to type-A heterocycles, we will now examine the synthesis of biologically active pyrazino[1,2-*a*]indoles which will be classified by their biological activities.

### 2.2. Biologically Active Pyrazino[1,2-a]indoles

#### 2.2.1. Neuropsychiatric Properties

**Scheme 3:**
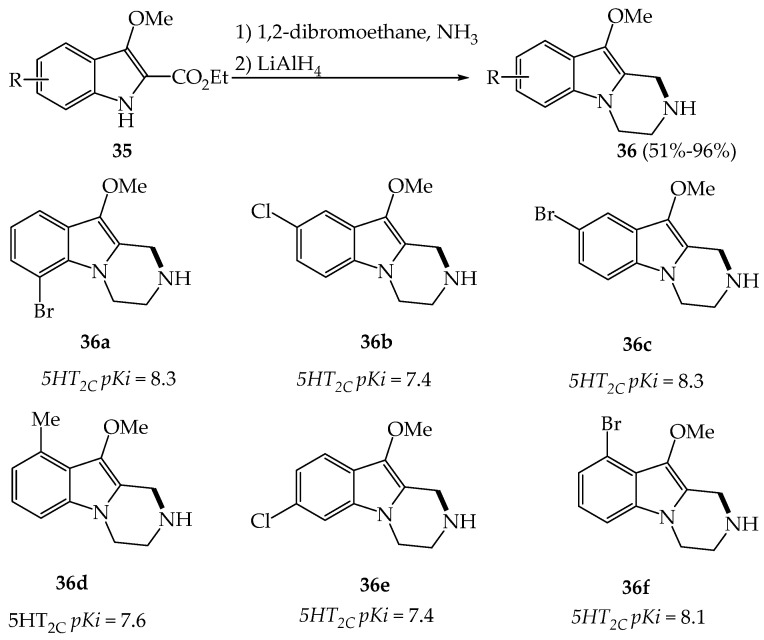
Synthesis of tetrahydro-pyrazinoindoles **36** and their binding data at the 5HT_2C_ receptor subtype.

Bos et al., in a program dedicated to the discovery of novel drugs for the treatment of neuropsychiatric disorders, synthesized a variety of pyrazino[1,2-*a*]indoles **36a–f** which were found as partial agonist ligands at the 5HT_2C_ receptor (Scheme 3) [25].

Pyrazinoindole derivatives **36a–f** were prepared according to a *N*-alkylation/cyclization/reduction sequence of indoles **35** having an ester function on C2 [25]. Pyrazinoindoles **36** were found to be partial agonists at the 5HT_2C_ receptor subtype binding with a higher affinity than for 5HT_2A_ receptors. Best affinities for 5HT_2C_ receptor were observed for 10-methoxy-pyrazinoindoles having on 6, 7, 8 or 9-position of the A-ring bulky atoms (F < Me < Cl < Br). In animals, **36d** showed a 30-fold selectivity for 5HT_2C_ receptors compared to 5HT_2A_ receptors and an only 3-fold selectivity compared to 5HT_1A_ receptors. In vivo results (rats and monkeys) also demonstrated that pyrazinoindole **36d** had a promising therapeutic potential for the treatment of various psychiatric disorders, such as obsessive-compulsive disorders, panic anxiety or depression.

**Figure 2:**
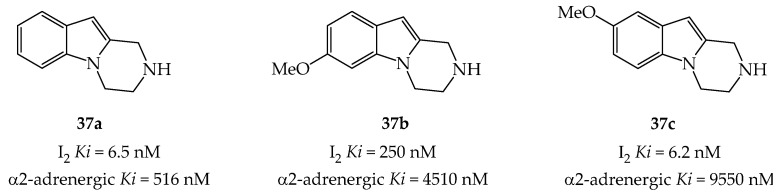
Tetrahydro-pyrazinoindoles **37** and their binding data at I_2_ and α-adrenergic receptors.

Imidazoline receptors exist in two forms, I_1_ and I_2_, for which there are very few ligands that are selective for one of the two forms. As a result, it is very difficult to assign a well-defined role to them even though I_2_ receptors have been described as involved in a variety of CNS disorders. Tetrahydro-pyrazinoindoles **37a–c** were evaluated by Glennon group for their potential as I_2_ imidazoline receptor ligands [26] due to their resemblance to β-carbolines [27] and imidazo-pyridoindoles (Figure 2) [28].

Remarkably, 8-methoxypyrazinoindole **37c** binds to I_2_ receptors with high affinity (*Ki* = 6.2 nM) and has a 1500-fold selectivity for I_2_ receptors compared to α2-adrenergic receptors (*Ki* = 9550 nM) and a 1000-fold selectivity for I_1_ receptors. A similar high selectivity for **37c** was also observed towards I_2_ receptors compared to serotonin 5HT_2A_ and 5HT_2C_ receptors.

**Scheme 4:**
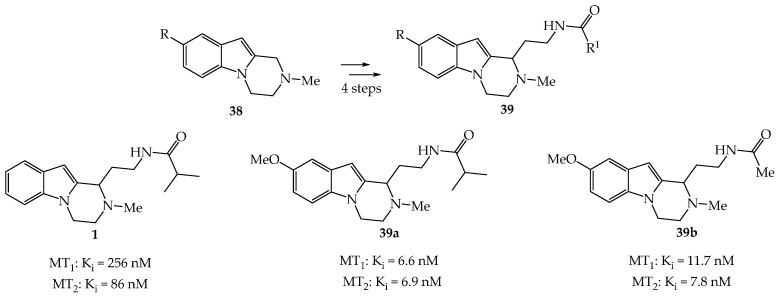
Synthesis of tetrahydro-pyrazinoindoles **1** and **39**.

Zlotos et al. synthesized a series of C1-substituted tetrahydro-pyrazinoindoles **1** and **39** as novel potent melatoninergic ligands from **38** in 4 steps (Scheme 4) [3].

The affinity of pyrazinoindoles **1**, **39a**,**b** for human MT1 and MT2 melatonin receptors in Chinese Hamster Ovary (CHO) cells was measured by competition binding analysis using 2-[^125^I]-iodomelatonin. The most active compound **39a** was found to be an interesting ligand for MT1 and MT2 receptors with excellent affinity, but with no subtype selectivity (MT1: Ki = 6.6 nM; MT2: Ki = 6.9 nM, respectively). This tetrahydro-pyrazinoindole compound was found to be a partial agonist at MT_1_ receptors and possessed no intrinsic activity at MT_2_ receptors. It is noteworthy that the treatment of pyrazinoindole 7 (R = H) with MeI gave the corresponding *N*-dimethyliodonium salt which was found to displace [^3^H]-cytisine from the nicotinic binding sites on rat cerebral cortex and was revealed to be a nicotinic agonist ligand [29].

#### 2.2.2. Auto-Immune Properties

**Scheme 5:**
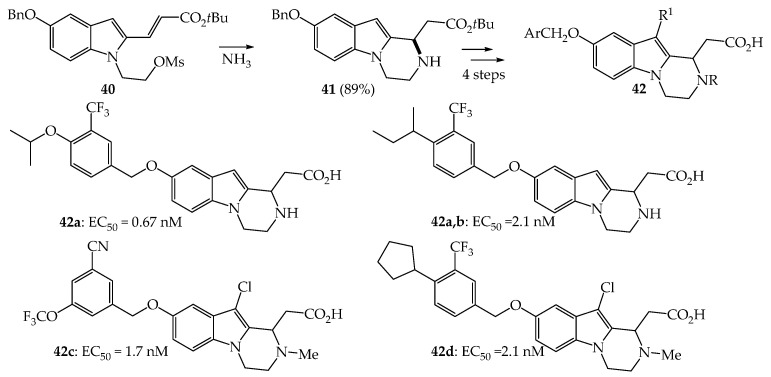
Synthesis of tetrahydro-pyrazinoindole **41** and human S1P_1_ cAMP EC_50_ values of derivatives **42a–d**.

Among the C1-substituted pyrazinoindoles, we can cite the work of Buzard et al. who prepared a series of C3-tetrahydro-pyrazinoindoles **42** from the same precursor **41** resulting from an intramolecular Michael reaction carried out on mesylate **40** in the presence of NH_3_ (Scheme 5) [30]. In a previous work, Buzard et al. showed that some cyclopenta[*b*]indoles were very potent agonists of the sphingosine 1-phosphate (S1P_1_) receptor that could be used for the treatment of certain autoimmune diseases [31]. Due to the structural resemblance to these indoles, a series of tricyclic analogues (pyridoindoles, oxazinoindoles and pyrazinoindoles) were designed, synthesized, and evaluated. Pyridoindoles proved to be the most promising compounds in this series of fused-indole compounds, even if pyrazinoindoles **42a–d**, prepared from **41** in four steps (*N*-Boc protection, *O*-debenzylation, *O*-functionalization with various benzyl chlorides and *t*-Butylester hydrolysis) showed interesting activities as S1P_1_ receptor agonists with nanomolar EC_50_ values. For the treatment of autoimmune disease as rheumatoid arthritis, Hill et al. synthesized a pyrazinoindole derivative having on C10 a substituted maleimide nucleus which was unfortunately found to be poorly active as protein kinase C inhibitor (IC_50_ = 540 nM) [32].

#### 2.2.3. Anti-Bacterial and Anti-Fungal Properties

A series of 15 pyrazinoindoles **43** were prepared according to Refs. [17,18] (see Scheme 2) by Verma group and evaluated for their anti-bacterial properties (Table 1) [33]. The in vitro antibacterial activity was evaluated by disc diffusion assay (DDA) using pathogenic strains of *Staphylococcus aureus*, *Salmonella typhi*, *Streptomyces thermonitrificans*, *Pseudomonas aeruginosa* and *Escherichia coli*. It was demonstrated that **43a** was only active on *P. aeruginosa* and, similarly, a significant activity on *P. aeruginosa* and *S. thermonitrificans* was noticed with **43b**. Pyrazinoindoles **43c–e** were found to be active against all tested strains but with a relatively modest efficacy when compared with gentamycin. From these results, it seems that the presence of substituents on the nitrogen atom of pyrazinoindoles is deleterious for a satisfactory anti-bacterial activity. Pyrazinoindoles having an aromatic C-ring were not active against all tested strains.

Tetrahydro-pyrazinoindoles **43** were also evaluated for their anti-fungal activity against *Aspergillus flavus*, *Aspergillus fumigatus*, *Aspergillus. niger* and *Candida albicans* (Table 2) [34]. The anti-*Aspergillus* activity was evaluated by disc diffusion assay (DDA) and the anti-*Candida* activity was investigated by microbroth dilution assay. The more active tetrahydro-pyrazinoindoles **43** presented in Table 2 displayed a mild to moderate anti-fungal activity, even if these pyrazinoindoles were found to be, in vitro, less cytotoxic than Amphotericin B when used at high concentrations. SARs with compounds **43** were similar for both anti-bacterial and anti-fungal activities.

#### 2.2.4. Anti-Arrhythmic, Anti-Lipolytic, Neuro- and Cardio-Protective Properties

**Scheme 6:**
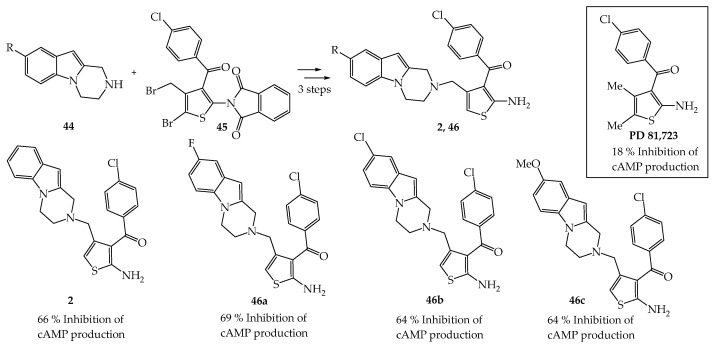
Synthesis of tetrahydro-pyrazinoindoles **2**, **46a–c** and effect in cAMP assay in hA_1_CHO cells.

In a program dedicated to the discovery of ligands able to activate the A_1_AR adenosine receptor, Romagnoli et al. proposed some derivatizations on PD81,723, an allosteric modulator acting at the A_1_AR receptor, enhancing the functional effects of adenosine receptor subtype (Scheme 6) [4].

Thus, pyrazinoindoles **2**, **46a–d** were synthesized from dibromothiophene **45** and 8-substituted pyrazinoindoles **44** in 3 steps (S_N_2 reaction, debromination, phthalimide hydrolysis). Pyrazinoindoles **2**, **46a–c** were next evaluated in a functional assay for their ability to inhibit forskolin stimulated cAMP production via the hA_1_-AR in intact Chinese hamster ovary (CHO) cells. The four pyrazinoindoles **2**, **46a–c** were found to be significantly more active than the reference PD 81,723. The best compound 8-fluorated pyrazinoindole **46a** inhibited the percentage of cAMP production by 69% vs. 18% for PD 81,723. It was also shown that these derivatives significantly inhibited antagonist binding at the hA_1_AR, hA_2_AR or hA_3_AR receptors.

#### 2.2.5. Anti-Cancer Properties

**Scheme 7:**
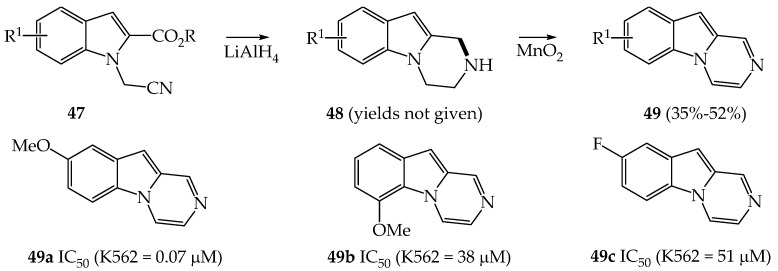
Synthesis and anti-cancer properties of a selection of pyrazinoindoles **49a–c**.

Romagnoli et al. studied in 2009 the antiproliferative properties of a series of pyrazinoindoles **17** which were prepared from the reduction/cyclization of *N*-cyanomethyl derivatives **47** followed by an oxidation reaction using MnO_2_ (Scheme 7) [35].

It was shown that pyrazinoindole **49a** was the more cytotoxic derivative against human leukemia K562 cancer cells with a promising IC_50_ value of 0.07 μM. However, this strong cytotoxicity was not observed in other cell lines such as murine leukemia (L1210), murine mammary carcinoma (FM3A), human T-lymphoblastoid (Molt/4 and CEM) and human cervical carcinoma (HeLa) cells with IC_50_ values superior to 20 μM.

**Scheme 8:**
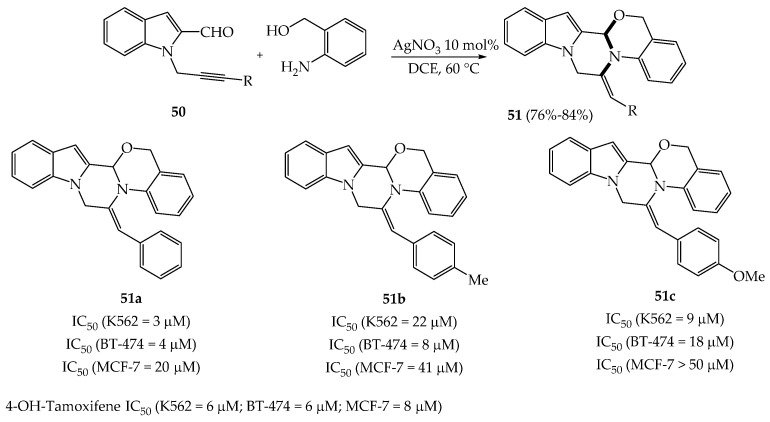
Synthesis of pyrazinoindoles **51** and their IC_50_ values against three human cancer cell lines.

In view of preparing pyrazinoindoles **51** as anti-cancer agents, Kumar et al. mixed *N*-propargyl indoles **50** having an aldehyde function on C2 with (2-aminophenyl)methanol derivatives in the presence of a catalytic amount of AgNO_3_ (Scheme 8) [36]. After the reaction of δ-alkynyl aldehydes and nucleophilic anilines, the alcohol function adds on the imine thus creating a second bond (C-O). The third bond creation (C-N) of this process occurs with the nitrogen atom of the imine which reacts with the alkyne triple bond activated by AgNO_3_ in a 6-*exo*-*dig* manner (76–84%).

Pyrazinoindoles **51** were next evaluated against 3 cancer cell lines (K562 leukemia cells, BT-474 human breast cells; MCF-7 breast cancer cells). As it can be seen in Scheme 8, the more cytotoxic compound was **51a** against K562 and BT-474 cancer cells. This pyrazinoindole was significantly more active than 4OH-tamoxifene, used as reference compound, against K562 and BT-474 cells but displayed a lower IC_50_ value against MCF-7 cancer cells. This result is interesting as **51a** exhibited maximum cytotoxicity in p53-deficient cell lines K562 and BT-474 cells but not in p53 wildtype MCF-7 cells. It would certainly be interesting to perform SARs on these structures and to evaluate them on a panel of human cancer lines resistant to the usual treatments.

After discussing the syntheses of pyrazinoindoles of type A and their biological activities, we will now detail the access to pyrazinoindol-1-ones B which have been more studied than pyrazinoindoles, probably because they are active on a larger number of biological targets.

## 3. Pyrazinoindol-1-Ones B: Synthesis and Biological Properties

### 3.1. Recent Synthetic Approaches to Variously Substituted Pyrazinoindol-1-Ones of Type B

**Scheme 9:**
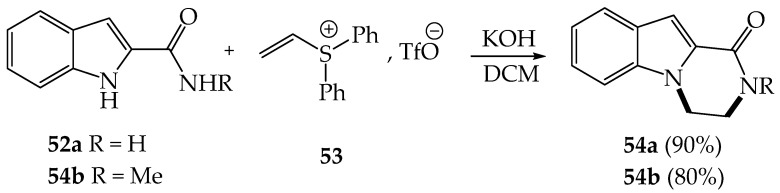
Synthesis of pyrazinoindolones **54a**,**b**.

One of the easiest methods to prepare tetrahydro-pyrazinoindol-1-one derivatives **54** was proposed by Chen and Xiao group in 2011 [37]. In the presence of vinylsulfonium salt **53**, the authors showed that variety of (1*H*-indol-2-yl)methanols were transformed with high yields into corresponding oxazinoindoles usable in medicinal chemistry [38]. It was next demonstrated that this easy-to-implement process was efficiently transposed to the synthesis of tetrahydro-pyrazinoindoles **54a**,**b** by replacing (1*H*-indol-2-yl)methanols, as nucleophiles, by indole-2-carboxamides **52a**,**b** (Scheme 9). The reaction proceeds via a Michael addition of the indole nitrogen anion on the electrophilic sulfonium salt followed by a S_N_2 substitution of the amide group, after prototropy, to give the expected pyrazino[1,2-*a*]indol-1-ones **54a**,**b** in excellent yields together with Ph_2_S.

**Scheme 10:**
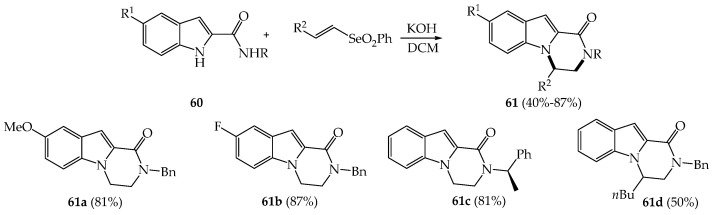
Synthesis of pyrazinoindolones **61** and selected examples**.**

The Michael reaction was also applied few years after by Bagnoli et al. under same conditions using secondary amides and vinylselenones (Scheme 10) [39].

As it can be seen with **61a** and **61b**, electron-donating and electron-withdrawing groups are welcome on the indole nucleus. It should be noticed that pyrazinoindoles having on C4 alkyl substituents (hexyl, butyl, methyl) were obtained in moderate to good yields (40–70%, e.g., **61d** 50%), probably due to steric hindrance considerations. Lastly, the reactivity of primary amides with vinylselenones was not studied under these conditions in this work.

**Scheme 11:**
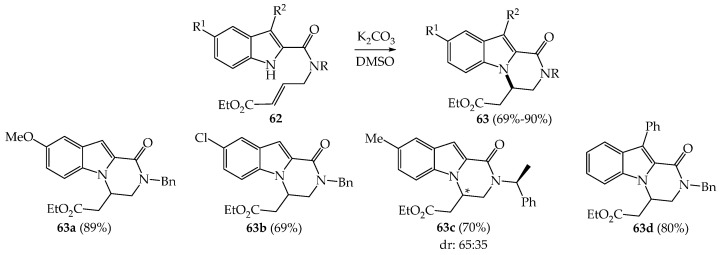
Synthesis of pyrazinoindolones **63** and selected examples.

Another aza-Michael version was proposed by the team of Bandini and Umani-Ronchi who considered the intramolecular cyclization of compounds **62** to give pyrazinoindol-1-ones **63** (Scheme 11) [40].

After a base and solvent screening study, it was demonstrated that the best combination is usage of K_2_CO_3_ (10 mol%) and DMSO as the solvent. Electron-donating and electron-withdrawing substituents on the indole led to desired pyrazinoindoles with comparable yields. The presence of aromatic substituents (phenyl, naphtyl) on the C3 position of indoles **62** did not affect the outcome of the cyclization reaction (e.g., **63d** 80%). Finally, enantiometric pure acrylate containing a (*S*)-phenylethylamine unit gave the expected pyrazinoindole **63c** in a good yield but with a modest diastereoselectivity.

**Scheme 12:**
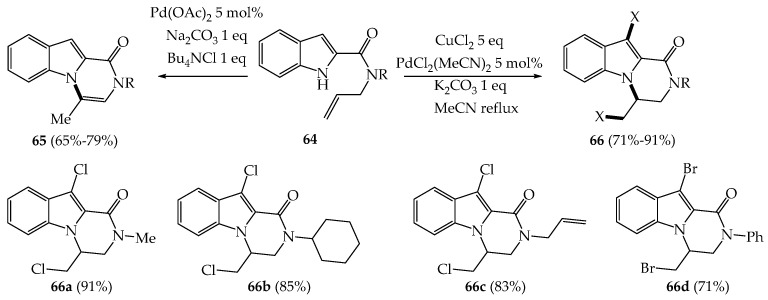
Synthesis of pyrazinoindolones **65** and **66** and selected examples.

In addition to Michael addition reactions giving access to pyrazinoindolones, methods using catalytic amounts of organopalladium catalyst have been described to prepare these substrates. For example, the group of Broggini studied the cyclization reactions of a variety of indoylallylamines **64** in the presence of PdCl_2_(MeCN)_2_ and CuCl_2_ [41] to produce pyrazinoindolones **66** or with Pd(OAc)_2_, Na_2_CO_3_, and Bu_4_NCl to give pyrazinoindolones **65** [42] (Scheme 12).

**Scheme 13:**
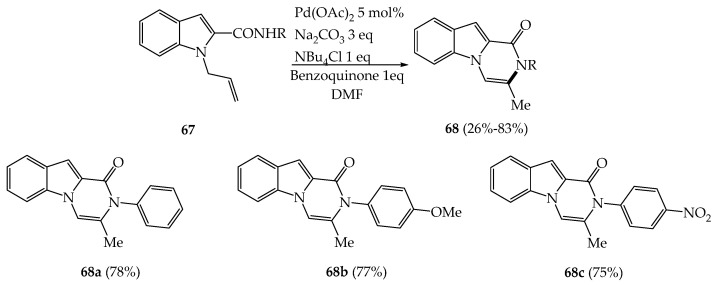
Synthesis of pyrazinoindolones **68** and selected examples.

The reaction producing **66** starts by the halogenation of the indoyl C3-position by CuX_2_ without involvement of the palladium catalyst. Then, an amino-palladation of the olefins π-bond occurs followed by a chlorine transfer on the Pd-species to give pyrazinoindolones **66** rather than β-hydride elimination products. This domino process, which allows the synthesis of di-halogenated pyrazinoindolones in very good yields, seems to be very suitable for the synthesis of complex and functionalized pyrazinoindolones. Broggini et al. also reported the synthesis of pyrazinoindolones **68** by using palladium-catalyzed cyclization of *N*-allylindoles **67** in the presence of Pd(OAc)_2_ as catalyst, Na_2_CO_3_ as base, benzoquinone as oxidant, and *tetra*-butyl ammonium chloride as additive in DMF (Scheme 13) [43].

**Scheme 14:**
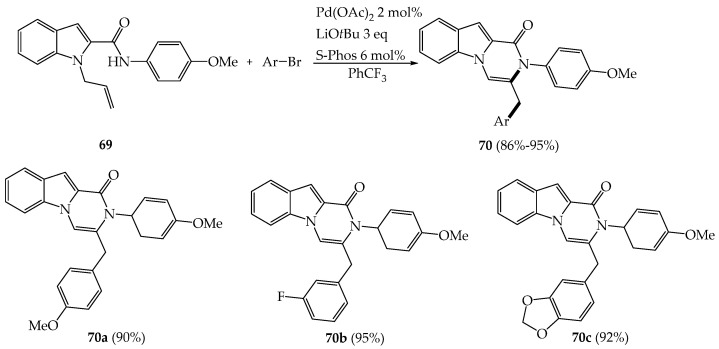
Synthesis of pyrazinoindolones **70** and selected examples.

Wolfe et al. proposed later an extension of this amino-palladation method by adding aromatic bromides to the re-examined reaction medium (Scheme 14) [44]. In this process, the catalytic cycle is initiated by oxidative addition of the Ar-Br on Pd(0) and, the resulting Pd(II) species coordinated the alkene double bond. After deprotonation of the amide, an amino-palladation occurred to give, after reductive elimination, **70** and regeneration of the Pd(0) catalyst.

**Scheme 15:**
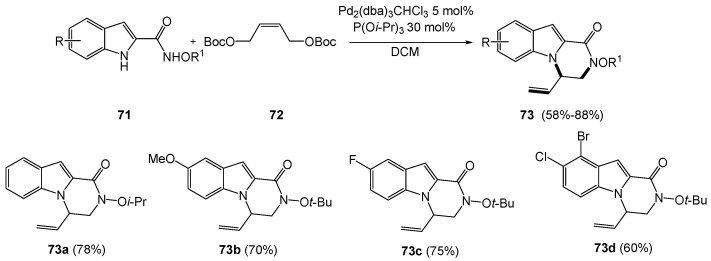
Synthesis of pyrazinoindolones **73** and selected examples.

Laliberté et al. prepared a series of pyrazinoindolones **73** with good yields arising from the Pd-coupling reaction between indole-hydroxamates **71** and electrophilic (*Z*)-but-2-ene (1,4-*bis*carbonate) **72** (Scheme 15) [45].

Electron-rich and electron-poor substituents are well tolerated at each position of the indole ring and gave the expected pyrazinoindoles in comparable yields. The R^1^ substituent of the hydroxamate was studied and when R^1^ = Bn, *i*-Pr, and *t-*Bu, the pyrazinoindolones were obtained in 72–81% yields, whereas with R^1^ = Me, the reaction is somewhat less efficient (58%). It would be interesting to study a chiral version of this reaction using suitable ligands to observe their effects on enantioselectivity.

**Scheme 16:**
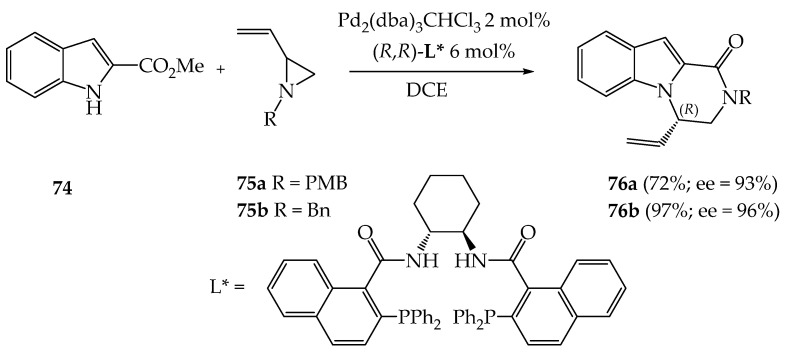
Synthesis of optically active pyrazinoindolones **34a**,**b**.

A chiral elegant access to various nitrogen-containing heterocycles was proposed by B. Trost during the transformation of vinyl aziridines with indoles and pyrroles (Scheme 16) [46]. Among the heterocycles evaluated as nucleophiles (pyrroles and indoles) in the presence of vinyl aziridines, it was showed that the use of Pd_2_(dba)_3_⋅CHCl_3_ with the chiral ligand L* in dichloroethane (DCE) provided access to the *N*-alkylation products in excellent yields and enantiomeric excesses in a regioselective manner. When these experimental conditions were applied to indolyl-methylcarboxylate **74** in the presence of vinylaziridines **75a**,**b**, pyrazinoindolones **76a**,**b** were obtained in good yields with excellent enantiomeric excess after opening of the aziridine followed by cyclization.

**Scheme 17:**
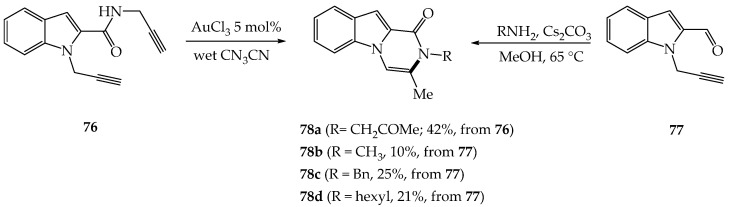
Synthesis of pyrazinoindolone **78a–d**.

A single example of gold-promoted cyclization of *N*-propargylamidolindole **76** giving pyrazinoindolone **78a** was proposed by A. Padwa (Scheme 17) [47]. In this reaction, the gold-catalyst (5 mol%) first activates the alkyne triple bond which is then attacked by the nucleophilic amide group. An additional hydration of the other triple bond could occur by re-using the gold-catalyst, to promote pyrazinoindolone **78a**. It should be noted that it is also possible to access with low yields to *N-*substituted-3-methyl derivatives **78b–d** by mixing *N*-propargyl-indole-2-carbaldehyde **77** in the presence of amines (MeNH_2_, BnNH_2_ and hexNH_2_) as nucleophiles and Cs_2_CO_3_ as a base without the need of any metal. The mechanism of this cyclization was discussed in detail by the authors [48].

**Scheme 18:**
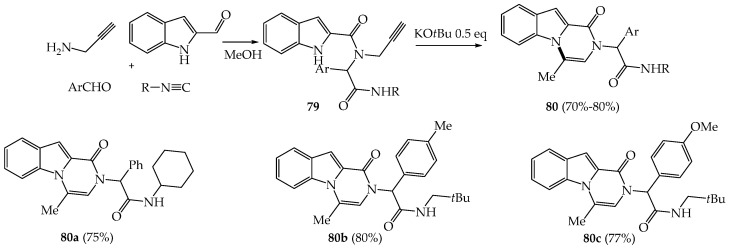
Synthesis of pyrazinoindolones **80** and selected examples.

The last example of this section concerns the synthesis of pyrazinoindolones **80** obtained in two steps from an Ugi condensation followed by a base-mediated cyclization on Ugi intermediates **79** (Scheme 18) [49].

After this overview of the synthetic routes to pyrazinoindolones published from a methodological point of view, we will now list the main accesses to biologically active pyrazinoindolones.

### 3.2. Biologically Active Pyrazino[1,2-a]indol-1-ones

#### 3.2.1. Anti-Cancer Properties

**Scheme 19:**
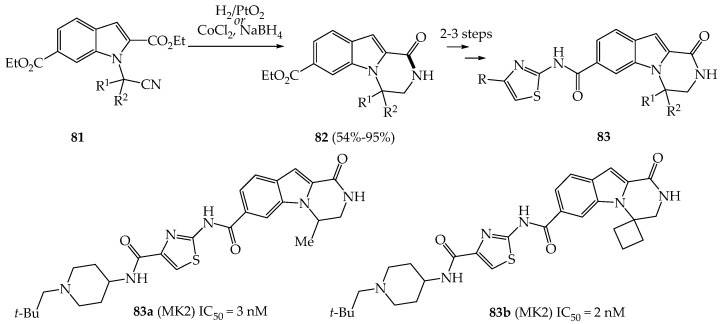
Synthesis of pyrazinoindolones **83** as MAPK2 inhibitors.

MAPK-activated protein kinase 2 (MAPK2), a serine/threonine-protein kinase known as the best understood downstream partner of p38 MAP kinase play an essential role in signal transduction pathways involved in cell proliferation, differentiation, and death. To prepare a series of MAPK Activated Protein Kinase 2 (MAPK2) inhibitors, Goldberg et al. used reductive conditions (H_2_/PtO_2_ or CoCl_2_/NaBH_4_) to indoyl-1-cyanomethyl derivatives **81** to afford, after cyclization, pyrazinoindolone platforms **82** (Scheme 19) [50]. Chemical modifications of the ethylester (3 steps) led to a series of twenty pyrazinoindolones **83** which were evaluated as MAPK2 inhibitors and best compounds are presented in Scheme 19. Pyrazinoindolones **83a** and **83b** were found to provide best combination of molecular, cellular, and physicochemical properties with nanomolar IC_50_ values. Pyrazinoindolone **83b** was also found to have a negligible effect on a wide range of other kinases, showing selectivity, and its efficacy was also demonstrated through various in vitro and in vivo tests.

**Scheme 20:**
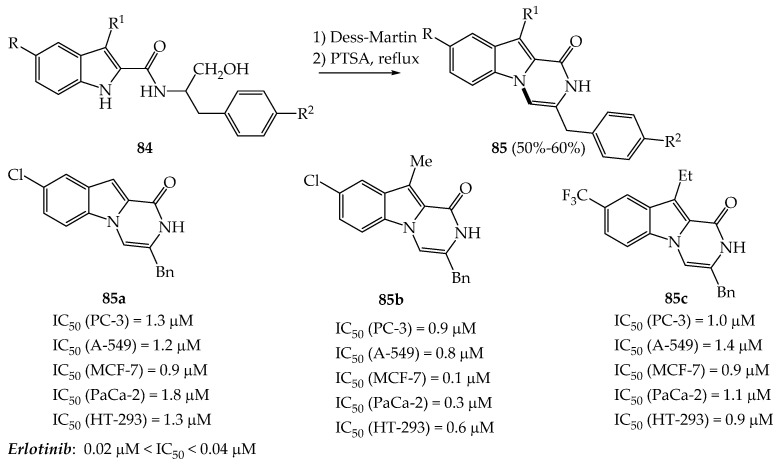
Synthesis and evaluation of pyrazinoindolones **85** as anti-cancer agents.

To counteract the deleterious effects of free radicals on DNA, which cause cancers, Bukhari et al. recently prepared and evaluated a small library of pyrazinoindoles **85** having on C3 benzyl substituents (Scheme 20) [51]. Pyrazinoindolones **85** which were found to be nontoxic against human mammary gland epithelial cells displayed a micromolar level of cytotoxicity against PC-3 prostate cancer cells, MCF-7 breast cancer cells, PaCa-2 pancreatic carcinoma cells, A-549 epithelial cancer cells and HT-29 colon cancer cells. It was shown that **85a–c** exhibited a low influence on tubulin assembly but inhibited EGFR with IC_50_ values from 1.7 to 3.9 μM. These compounds were also found to be inhibitors of reactive oxygen species (ROS) and showed noticeable antioxidant activity. Histopathological and immunohistochemical studies showed that, when chlorpyrifos, a ROS enhancer, was associated to pyrazinoindolone **85b** on the testis of male mice, testicular damage was significantly decreased.

**Scheme 21:**
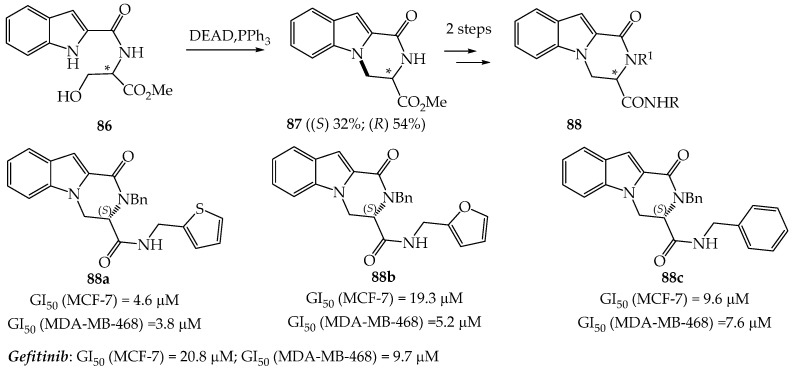
Synthesis and evaluation of optically active 3-substituted pyrazinoindolones **88**.

Kwak group synthesized a wide range of enantiopure (*R*)- and (*S*)-3-substituted pyrazinoindolones **88** from an intramolecular Mitsunobu cyclization of **86** using DEAD, PPh_3_ in THF (Scheme 21) [52].

Compounds **88** were evaluated for their anti-proliferative activity on MCF-7 breast cancer cells and on triple negative MDA-MB-468 breast cancer cells. It was showed that the stereochemistry on C3 has not a significant impact on the cytotoxicity. Interestingly, it was observed that the most cytotoxic compounds **88a–c** presented in Scheme 21 were more effective than the reference compound gefinitib. Furthermore, when **88b** was combined with gefinitib, a synergistic effect was observed, and the level of cytotoxicity was increased in the MDA-MB-468 cell line. Using Western blot analysis, the authors confirmed that best pyrazinoindolones inhibited phosphorylation of Akt signaling pathway in the MDA-MB-468 cell line.

**Scheme 22:**
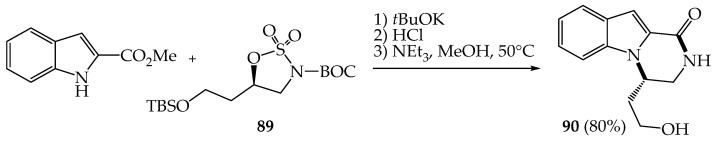
Synthesis and evaluation of optically active pyrazinoindolone **48**.

In a work dedicated on the synthesis of Longamide B analogs, Fujimoto et al. prepared and evaluated C4-substituted pyrazinoindolone **90** as a potential inhibitor of indoleamine 2,3-dioxygenase 1 (IDO1, an enzyme overexpressed in certain cancers such as colon and stomach cancers (Scheme 22) [53].

Compound **90** resulted from a nucleophilic reaction of methyl indole-carboxylate on **89** followed by protecting group removal and cyclization (80%; overall yield). The IDO1 inhibitory activity was evaluated at a concentration of 1 mM but, unfortunately, pyrazinoindolone **90** showed a poor IDO1 inhibitory activity (20%).

**Scheme 23:**
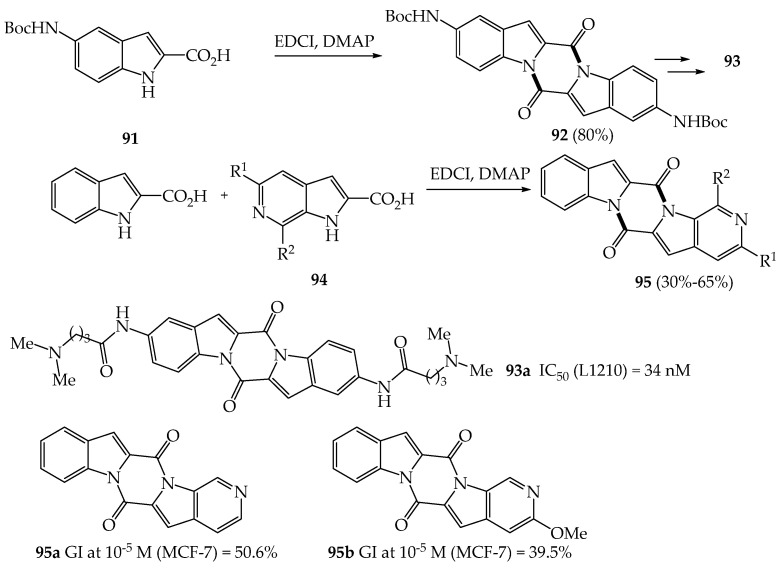
Synthesis and cytotoxic properties of symmetrical and unsymmetrical diketopiperazines **95** and **95**.

Boger et al. serendipitously prepared symmetrical diketopiperazine **92** by reacting indole-2-carboxylic acid **49** with 1-ethyl-3-(3-dimethylaminopropyl)carbodiimide (EDCI) in DMAP (Scheme 23) [54]. After Boc-deprotection of **92**, a series of lactam derivatives was synthesized and evaluated against a mouse lymphocytic leukemia cell line (L1210) and the best compound **93a** was found to be highly cytotoxic with a IC_50_ value of 34 nM.

A few years later from the above-mentioned study, Montalbano et al., with the same reaction conditions, has synthesized various unsymmetrical diketopiperazines **95** with variable yields. Unfortunately, these derivatives exhibited modest cytotoxicity against a panel of 60 human cancer cell lines [55]. The group of Vigusin and Moody also prepared various pyrazinoindole-1,4-diones as gliotoxin analogues from the reaction of indole-2-carboxylic acid with sarcosine ethyl ester hydrochloride in the presence of EDCI (Scheme not shown). Unfortunately, pyrazinoindole-dione [56] compounds were found to be poorly active as inhibitors of farnesyltransferase (FTase) and geranylgeranyltransferase (GGTase I) [57].

**Scheme 24:**
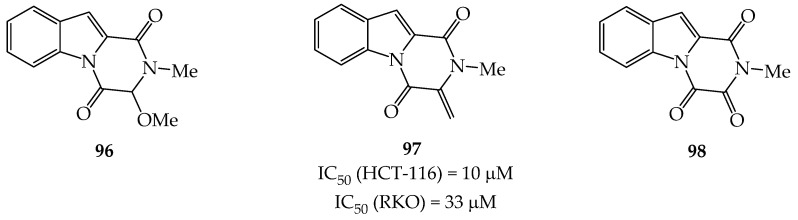
Secondary metabolites **96**–**98** from the marine fungus *Neosartorya pseudofischeri.*

Diketopiperazines have been isolated as secondary metabolites from the marine fungus *Neosartorya pseudofischeri* (Scheme 24) [58]. Of the five pyrazinoindole-diones isolated, only compound **97** was found to be cytotoxic against the human colon cancer lines HCT116 and RKO with IC_50_ values of 10 and 33 μM, respectively. Moreover, **97** was found to possess anti-bacterial activity and inhibited the growth of *Staphylococcus aureus* ATCC29213 and R3708 with MIC values of 283 and 70 μM.

#### 3.2.2. Anti-Infectious Properties

**Scheme 25:**
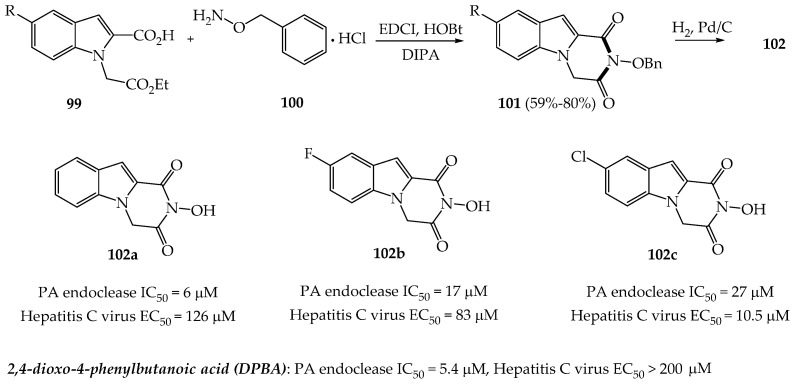
Synthesis and evaluation of a selection of hydroxyimides **102** as antiviral compounds.

Zoidis et al., in a program dedicated to the synthesis of novel indole-flutimide having activity against influenza PA endonuclease and Hepatitis C virus, prepared and evaluated five novel *N*-hydroxyimides **102** (Scheme 25) [59]. Intermediate *O*-benzyl-compounds **101** were easily obtained from the condensation of indoles **99** with *O*-benzyl hydroxylamine **100** in the presence of HOBt and EDCI. The authors demonstrated that incorporating the 2,6-diketopiperazine moiety of flutamide into the pharmacophore ring of indole, the resulting hydroxyimides **102** displayed potent inhibitory against influenza PA endonuclease with micromolar IC_50_ values. Compound **102a** was found to be as potent as 2,4-dioxo-4-phenylbutanoic acid (DPBA), a known PA_N_ inhibitor used as reference. Interestingly, hydroxyimide **102c** showed notable anti-hepatitis C virus (HCV) activity (EC_50_ = 10.5 µM) in Huh5-2 cell line harboring the firefly luciferase-expressing subgenomic replicon of the HCV genotype 1b Con1 strain. These compounds were next evaluated by Tavis group as possible inhibitors of Hepatitis B virus but, unfortunately, were found to be ineffective [60].

**Scheme 26:**
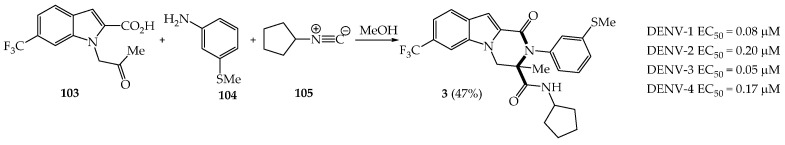
Synthesis of a dengue inhibitor **3**.

In 2005, Ivachtchenko and co-workers proposed an efficient three-component synthesis of 3-substituted pyrazinoindoles from a Ugi condensation of ketoacids with anilines and isonitriles [61]. This protocol was used a few years later by Yokokawa group in a program dedicated to the synthesis of dengue inhibitors, to prepare pyrazinoindolone **3** using ketoacid **103** with 3-thiomethylaniline **104** and isocyanide **105** (Scheme 26) [5].

After a chiral separation, each enantiomer of **3** were evaluated separately in an A549 cell-based flavivirus immunodetection (CFI) assay. A marked difference in EC_50_ values was observed between the (3*R*) and (3*S*) enantiomers highlighting the importance of chirality in compound **3** for this type of tropical disease. In all DENV 1–4 serotypes, (3*S*)-**3** was found to display very low EC_50_ values ranging from 0.01 to 0.09 μM and was found to be up to 50 times more effective than the (3*R*)-enantiomer.

#### 3.2.3. Neuropsychiatric Properties

**Scheme 27:**
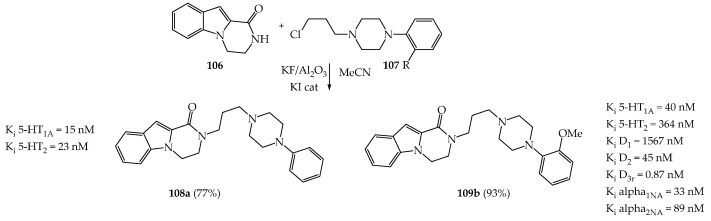
Synthesis of **108a**,**b** as 5-HT_1A_ and 5-HT_2_ ligands.

One of the oldest examples of biologically active pyrazinoindolone derivatives was reported in 1994 by Mokrosz et al. who synthetized a series of new antagonist ligands of 5-HT_1A_-, 5-HT_2_-, and D_2_-receptors (Scheme 27) [62].

A S*_N_*2 reaction between 3,4-dihydropyrazino[1,2-*a*]indol-1(2*H*)-one **106** and 1-aryl-4-chloropropylpiperazines **107** gave a rapid access to **108a** and **108b** with good yields (77% and 93%, respectively). As shown, pyrazinoindolones **108a** and **108b** displayed good affinities for 5-HT_1A_ receptor with Ki values of 15 and 40 nM. It was also shown that **108a** had a better affinity than **63b** on 5-HT_2_ receptor. However, compound **108a** did not show significant selectivity for 5-HT_1A_/5-HT_2_, and chemical transformations could perhaps overcome this problem. A decade after, Campiani et al. re-studied effects of pyrazinoindolone **108b** on a variety of D_1_-_3_ dopamine, 5-HT_1A_ serotonin, and alpha-noradrenaline receptor subtypes [63]. It was demonstrated that **108b** showed D_3_ receptor sub-nanomolar affinity (K_iD3_ = 0.87 nM) with an excellent D_2_/D_3_ affinity ratio of 52 and a good D1/D3 affinity of 1801. This study demonstrated that pyrazinoindolone **108b** represented a very promising lead for the generation of novel D_2_/D_3_ receptor ligands potentially active against cocaine craving.

#### 3.2.4. Anti-Allergenic Properties

**Scheme 28:**
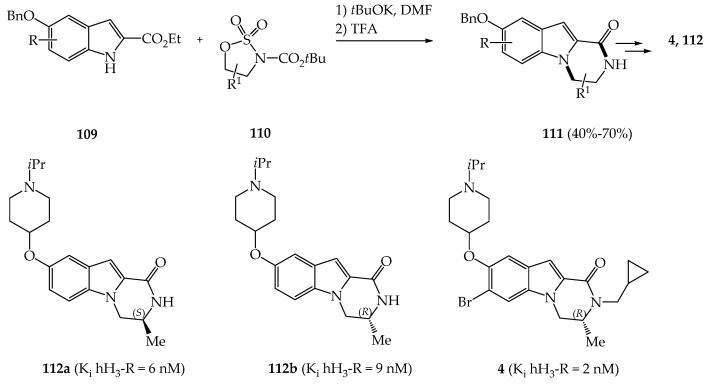
Synthesis of pyrazinoindolones **4**, 112, and affinity over histamine H3 receptor subtype.

The last example of this review concerns the synthesis and the biological properties of a series of substituted pyrazinoindolones **4** and **112** which were evaluated as histamine H_3_ receptor inverse agonists (Scheme 28) [6].

Alkylation of 5-benzyloxy-1*H*-indole-2-carboxylic acid ethyl ester **109** with various 2,2-dioxo[1–3]oxathiazolidine-3-carboxylic acid *tert*-butyl esters **110** gave pyrazinoindolones **111** in high yields. In case of chiral 5-substituted sulfamidates 110, the alkylation reaction proceeded under inversion of configuration with an enantiomeric excess of more than 98% ee. After adequate transformations (*O*-debenzylation followed by Mitsonobu reaction), all compounds were tested in a functional GTPgS assay and were characterized as potent full inverse agonists at the human H3 receptor (hH_3_-R) with K_i_ values at a nanomolar level. It was shown that the introduction of a methyl substituent was interesting on C3 rather than on C4, and the 3-Me (*S*) analogue **112a** displayed highest affinity with its enantiomer **112b**. In general, most compounds **112** which showed moderate affinity against hH_2_ and hH_4_ receptors were selective against hH_3_ receptor subtype. Oral administration in rat showed that compound **4** was tolerated with no significant side-effects, was well absorbed, and penetrated in brain successfully.

## 4. Conclusions

**Figure 3:**
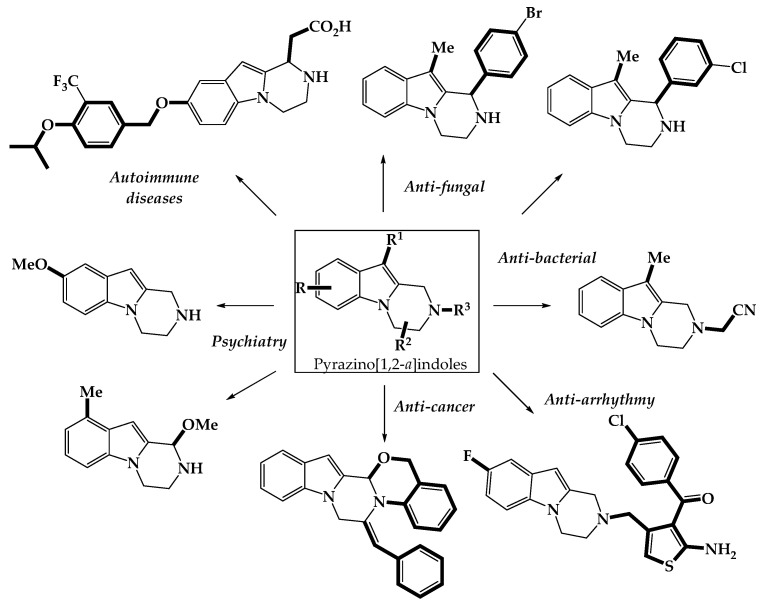
Overview of tetrahydro-pyrazino[1,2-*a*]indoles having biological properties.

In conclusion, it appears that the synthesis of pyrazinoindolones seems easier than that of their pyrazinoindole counterparts, probably because indole precursors bearing on C2 amides, esters or acid groups 2 are abundant and commercially available. Figure 3 highlights the various biological properties of tetrahydro-pyrazinoindoles, such as anti-fungal, anti-bacterial, anti-cancer, anti-arrhythmia, and others.

It would certainly be interesting to develop asymmetric pathways allowing access to C1-, C3- or C4-substituted compounds in an enantioselective manner to test their affinity and selectivity for the considered target(s).

**Figure 4:**
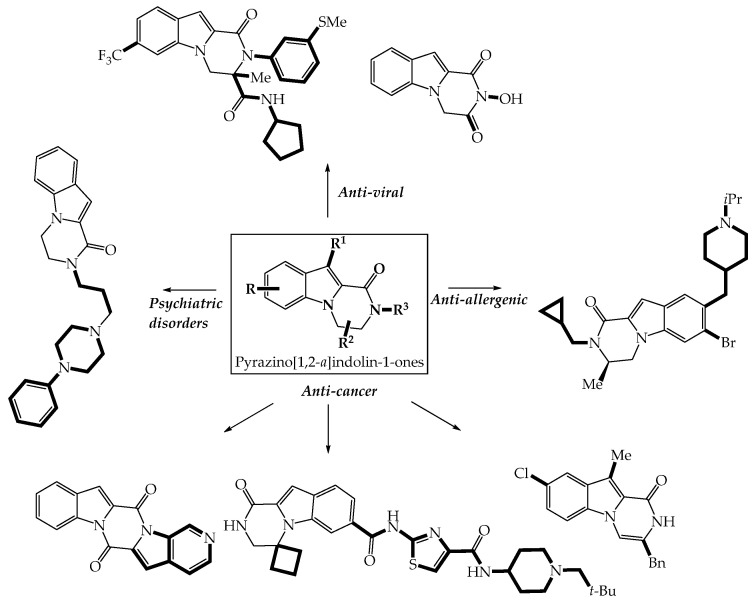
Overview of pyrazino[1,2-*a*]indol-1-ones having biological properties.

For pyrazinoindol-1-one derivatives whose biological activities are grouped in Figure 4, some of these compounds with an extremely simple chemical structure have been shown to be biologically very active, especially as anti-cancer agents with nanomolar level of cytotoxicity.

We now hope that all the observations reported in this review could open very interesting perspectives in medicinal chemistry (anti-cancer, anti-infectious, anti-allergenic, and others). These outlooks will quickly incite medicinal chemists to design new effective and selective ligands based on these types of chemical structures.

## Figures and Tables

**Table 1:**
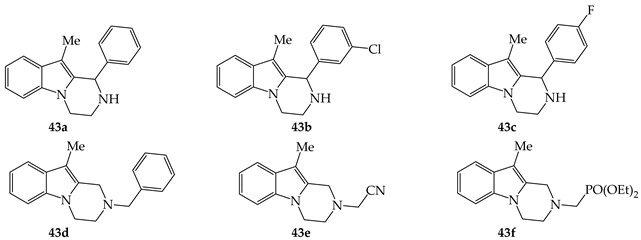
Anti-bacterial properties of tetrahydro-pyrazinoindoles **43a–f**.

Cpnd	DDA Minimum Inhibitory Concentrations (μg/disc)			
	*S. aureus*	*S. typhi*	*P. aeruginosa*	*S. thermonitrificans*
**43a**	-	-	3.75	-
**43b**	-	-	15	3.75
**43c**	30	30	30	7.5
**43d**	15	60	60	60
**43e**	15	30	60	30
**43f**	-	-	60	60
Gentamycin	1	1	0.5	1

**Table 2:**
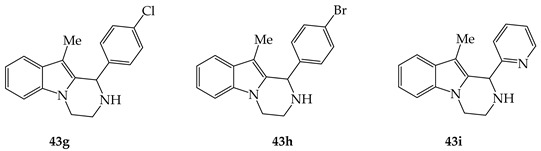
Anti-fungal properties of tetrahydro-pyrazinoindoles **43g–I** and **43c**.

Cpnd	DDA Minimum Inhibitory Concentrations (μg/disc)			
	*A. flavus*	*A. fumigatus*	*A. niger*	*C. albicans*
**43g**	11.7	5.8	11.7	15.6
**43h**	47	23	47	62.5
**43i**	187	94	187	125
**43c**	47	47	47	125
Gentamycin	1	1	0.5	1

## Data Availability

Data sharing not applicable.
